# Intratumoral hemorrhage, vessel density, and the inflammatory reaction contribute to volume increase of sporadic vestibular schwannomas

**DOI:** 10.1007/s00428-012-1236-9

**Published:** 2012-05-04

**Authors:** Maurits de Vries, Pancras C. W. Hogendoorn, Inge Briaire-de Bruyn, Martijn J. A. Malessy, Andel G. L. van der Mey

**Affiliations:** 1 Department of Pathology, Leiden University Medical Center, P.O. Box 9600, 2300 RC Leiden, The Netherlands; 2 Department of Neurosurgery, Leiden University Medical Center, P.O. Box 9600, 2300 RC Leiden, The Netherlands; 3 Department of Otolaryngology, Leiden University Medical Center, P.O. Box 9600, 2300 RC Leiden, The Netherlands

**Keywords:** Vestibular schwannoma, Neuropathology, Tumor biology

## Abstract

Vestibular schwannomas show a large variation in growth rate, making prediction and anticipation of tumor growth difficult. More accurate prediction of clinical behavior requires better understanding of tumor biological factors influencing tumor progression. Biological processes like intratumoral hemorrhage, cell proliferation, microvessel density, and inflammation were analyzed in order to determine their role in vestibular schwannoma development. Tumor specimens of 67 patients surgically treated for a histologically proven unilateral vestibular schwannoma were studied. Preoperative magnetic resonance imaging (MRI) scans were used to determine tumor size and to classify tumors as homogeneous, inhomogeneous, and cystic. Immunohistochemical studies evaluated cell proliferation (histone H3 and Ki-67), microvessel density (CD31), and inflammation (CD45 and CD68). Intratumoral hemorrhage was assessed by hemosiderin deposition. The expression patterns of these markers were compared with tumor size, tumor growth index, MRI appearance, patients’ age, and duration of symptoms. No relation between cell proliferation and clinical signs of tumor volume increase or MRI appearance was found. Intratumoral hemosiderin, microvessel density, and inflammation were significantly positively correlated with tumor size and the tumor growth index. Cystic and inhomogeneous tumors showed significantly more hemosiderin deposition than homogeneous tumors. The microvessel density was significantly higher in tumors with a high number of CD68-positive cells. The volume increase of vestibular schwannomas is not based on cell proliferation alone. Factors like intratumoral bleeding, (neo)vascularization, and intensity of the inflammatory reaction also influence tumor volume.

**Electronic supplementary material:**

The online version of this article (doi:10.1007/s00428-012-1236-9) contains supplementary material, which is available to authorized users.

## Introduction

Vestibular schwannomas (acoustic neuromas) are benign tumors originating from the myelin-forming Schwann cells of the vestibular branch of the eighth cranial nerve in the internal auditory canal or cerebellopontine angle. Clinically, these tumors grow slowly and progressively, eventually causing brainstem compression.

One of the problems in the treatment of these tumors is the large variation in the evolution of increase in volume in time. Why this is so variable remains largely unknown, but clinically, this makes prediction and anticipation of the evolution of symptoms difficult. Current therapeutic management is mainly based on symptoms and radiological assessment. Unfortunately, these indicators do not always correlate with actual tumor volume increase. More accurate prediction of tumor expansion rate could lead to more balanced decision-making regarding therapeutic actions when a vestibular schwannoma is diagnosed.

Accurate assessment of potential tumor volume increase requires better understanding of tumor biological (growth) factors and concurrent pathological events like cyst formation. To gain more insight in the role of these factors, we assessed the relationships between (immuno)histopathological markers, radiological observations on tumor appearance, and clinical growth of vestibular schwannomas.

One way for tumors to expand is by cell proliferation. A well-known proliferation marker is the Ki-67 antigen [1, 2], which is present during all phases of the cell cycle but is absent in noncycling cells. A less known indicator for proliferation is the phosphorylated histone H3 protein, which is expressed during mitosis [3–5]. Both these markers were included in this study.

In addition to proliferation, we investigated to what extent intratumoral hemorrhage, microvessel density, and the degree of inflammation are involved in tumor expansion. To quantify intratumoral hemorrhage, hemosiderin (i.e., iron) deposition was evaluated. The endothelial marker CD31 was used to measure microvessel density, and the degree of inflammation was determined by quantifying the number of leukocytes and macrophages through the expression of the markers CD45 and CD68, respectively.

## Material and methods

### Patients

From the vestibular schwannoma database at the Leiden University Medical Center, a total of 67 patients (26 males and 41 females) were identified. They involved a group of patients surgically treated for a histologically proven vestibular schwannoma from January 2000 to November 2005. The main criteria for surgical treatment were clinical symptoms (e.g., progressive or debilitating vertigo and hearing loss) and tumor size, both initial as well as in terms of progression over time. Patients’ personal preference was also of great importance in deciding if and when to apply treatment. Only cases of unilateral sporadic schwannomas were selected, patients diagnosed with NF2 were excluded.

### Tumor measurement

Information on radiological data was obtained from radiology records which are all composed according to specific clinical and scientific incentives. These standardized reports include measurement of the greatest tumor diameter according to the guidelines of the American Academy of Otolaryngology Head and Neck Surgery [6]. Additionally, the reports comprise evaluation of tumor density based on gadolinium-enhanced T1-weighted as well as T2-weighted scans. Depending on the presence of microcystic and or macrocystic components, tumors were classified as homogeneous, inhomogeneous, or cystic. All patient data were prospectively discussed at a multidisciplinary conference attended by representatives of the neurosurgery, radiology, radiotherapy, pathology, and otolaryngology department. Clinical and radiological data were cross checked before being entered in the vestibular schwannoma database (for the exact patient characteristics, including figures illustrating cystic, inhomogeneous, and homogeneous tumors, see Electronic supplementary material Figs. 1, 2, and 3).

### Immunohistochemistry

The immunohistochemical tests were conducted at the Department of Pathology of the Leiden University Medical Center. The tests were conducted on specimens preserved in formalin and stored in paraffin. All assessments were performed on tissue sections obtained from one single tumor block per tumor sample. Immunohistochemical reactions were performed according to standard laboratory methods [7]. In brief, heat-induced antigen retrieval was performed using microwave treatment of all sections after dewaxing and rehydration, followed by blocking of endogenous peroxidase with 3 % H_2_O_2_ in methanol. Incubation with primary antibodies was overnight (for sources, working dilutions, and positive controls used, see Table 1). Subsequently, sections were incubated with poly-HRP-antimouse/rabbit/rat conjugate. Visualization was carried out with a H_2_O_2_–diaminobenzidine solution. All washing procedures were performed in phosphate-buffered saline. Slides were counterstained with hematoxylin. In addition to the immunohistochemical stains, hemosiderin staining was performed.

**Table 1:** Antibody concentrations and positive control tissues used

Antibody	Supplier	Concentration	Positive control
Ki-67	DAKO	1:800	Tonsil
Histone H3	Comproscientific	1:3,200	Tonsil
CD31	DAKO	1:150	Tonsil
CD45	DAKO	1:4,000	Tonsil
CD68	DAKO	1:20,000	Tonsil

### Microscopic analysis

In order to obtain an overall impression of the staining pattern of each marker, all sections were first evaluated using a × 10 objective lens. Hemosiderin, CD45, and CD68 displayed an irregular/patchy staining pattern, making computer-assisted analysis less reliable. For this reason, further analysis of these markers was performed manually in a semiquantitative score. Per specimen, 10 randomly chosen fields of view (FOV) were evaluated at a × 40 objective lens. Scoring was independently assessed by two authors. The degree of staining was scored as follows: 0, absent; 1, mild; and 2, strong.

Staining for Ki67 and histone H3 showed a more homogeneous distribution, permitting the application of a computer-based image analysis method in order to determine the index of proliferation. In brief, of each specimen, five randomly chosen digital snapshots were taken. Positively stained nuclei were identified through spectrometry using a Leica DM4000B microscope fitted with a CRI Nuance spectral analyzer (Cambridge Research and Instrumentation, Inc., Woburn, MA, USA). Using Mirax software (Zeiss, Germany) and ImageJ (National Institutes of Health, Bethesda, MD, USA), the number of pixels representing positively stained nuclei was determined, as well as the number of pixels representing the total nuclear area in one FOV. The index of proliferation was calculated by dividing the number of immunopositive pixels by the number of pixels representing the total nuclear area. The mean value of the five snapshots was used for statistical analysis.

**Fig. 1:**
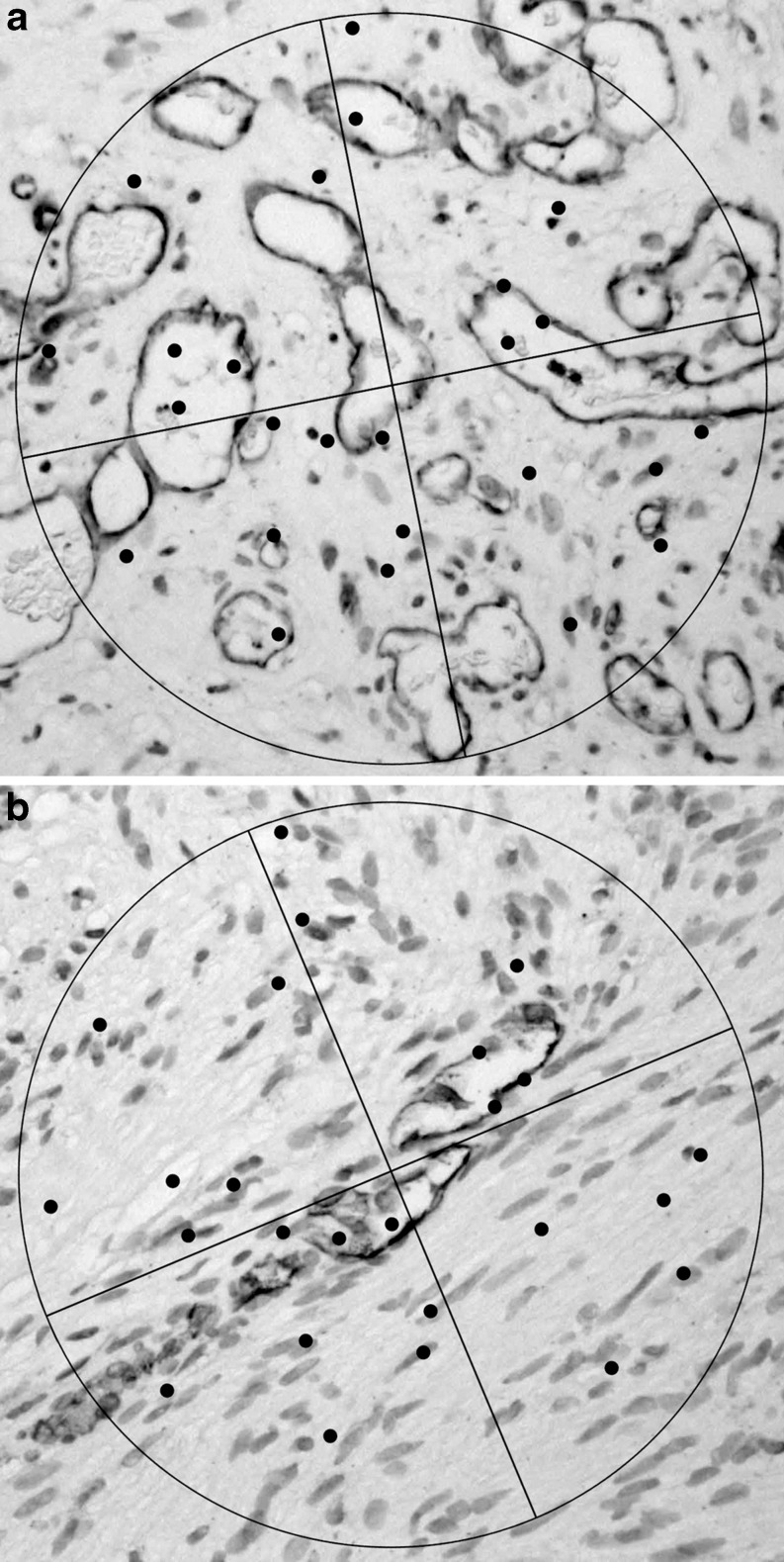
Examples of Chalkley counts in vascular hotspots, specimen **a** and **b** score 10 and 6, respectively (original magnification × 200)

Microvessel density was determined with the Chalkley point overlap morphometric technique, which allows for rapid analysis with a relatively low interobserver variability [8]. This method has been described in detail [9, 10]. In brief, CD31 stained sections were scanned for hot spots of high vascular density. Using an ocular grid with 25 random points, microvessel density was scored in these hot spots with a × 20 objective lens. The grid was oriented to permit the maximum number of points to hit the stained microvessels (Fig. 1). The Chalkley count was the mean of the maximum number of points hitting a microvessel in three hot spots per tumor specimen.

### Tumor growth index

Accurate measurement of tumor growth depends on serial magnetic resonance imaging (MRI) scanning over a longer period of time. Because only six patients in our selection underwent serial MRI scanning prior to surgery, this method of growth assessment could not be used in our study. We used the tumor growth index as a surrogate parameter. This index has been described earlier regarding vestibular schwannoma growth [11, 12]. Its basic assumption is that the age of onset of the tumor varies randomly. The tumor growth index is calculated by dividing the maximal tumor diameter by the age of the patient.

### Statistical analysis

For evaluation of the relationships between the tumor biological markers and parameters of clinical growth, the Spearman rank correlation test was used. The difference in hemosiderin deposition between cystic and inhomogeneous tumors versus homogeneous tumors was determined by the chi-square test. Tumor size of cystic and inhomogeneous tumors versus homogeneous tumors was compared with the unpaired *t* test. The relation between microvessel density and CD68 expression was evaluated using the one-way analysis of variance (ANOVA) and Scheffe test. For all statistical tests, *p* < 0.05 was considered as significant. All calculations were performed using SPSS Inc. software, version 17.0.

## Results

A total of 67 patients (age: range, 15–72 years; mean, 49.04 ± 14.06 years) was studied. Maximum tumor diameter varied between 5 and 50 mm (mean, 24.03 ± 11.52 mm) and the tumor growth index (which does not represent actual increase in size but a parameter for tumor growth rate) varied between 0.07 and 2.11 mm/year (mean, 0.56 ± 0.40 mm/year).

The mean value of the Ki-67 index in this studied group was 0.6 % (range, 0.1–1.8 %). For the histone H3 index, the mean value was 3.9 % (range, 0.4–15.5 %). No significant correlation was found between the Ki-67 or histone H3 index and maximal tumor diameter, tumor growth index, and radiologically measured tumor growth.

**Fig. 2:**
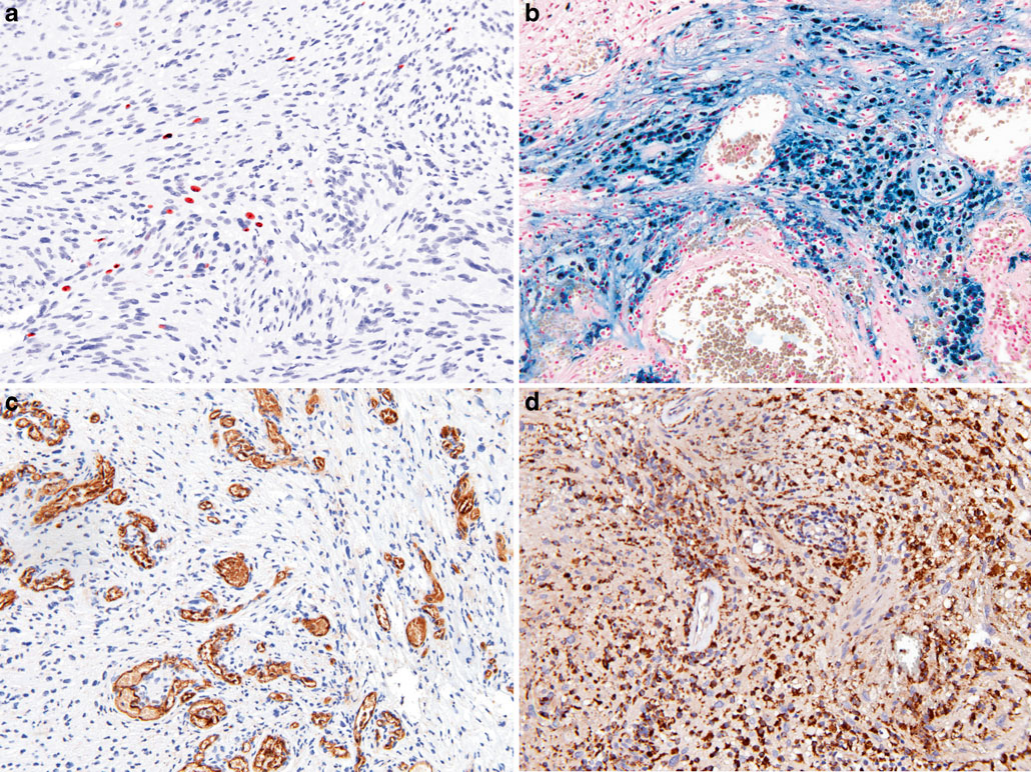
**a** Proliferating cells (Ki-67/histone H3), **b** intratumoral bleeding (hemosiderin), **c** high microvessel density (CD31), and **d** intratumoral inflammation (CD45/CD68) (original magnification × 400)

The number and distribution of hemosiderin-, CD45-, and CD68-positive cells in the tissue sections were very heterogeneous. Some specimens showed no staining at all, yet other specimens showed a substantial number of positive cells (Fig. 2). The mean Chalkley count was 9.59 (range, 3.3–23.0; SD, 3.70). The Spearman rank correlation test demonstrated several significant positive correlations between these markers (Table 2, for the corresponding scattergrams, see Electronic supplementary material Figs. 4, 5, and 6).

**Table 2:** Spearman’s correlation test for pathological markers and clinical characteristics

		Ki-67	Histone H3	CD45	CD68	Siderin^a^	MVD
Size	Correlation coefficient	0.129	0.326	0.333**	0.457**	0.392**	0.397**
	Significance (two-tailed)	0.301	0.120	0.006	0.000	0.001	0.001
TGI	Correlation coefficient	0.091	0.275	0.375**	0.482**	0.330**	0.373**
	Significance (two-tailed)	0.466	0.243	0.002	0.000	0.006	0.002
MVD	Correlation coefficient	0.140	0.240	0.281*	0.472**	0.363**	–
	Significance (two-tailed)	0.261	0.102	0.021	0.000	0.003	–

*TGI* tumor growth index, *MVD* microvessel density

^a^Hemosiderin

The number of hemosiderin-positive cells correlated with tumor size (*p* < 0.001; *r* = 0.39) and tumor growth index (*p* < 0.006; *r* = 0.33). CD45 expression correlated with tumor size (*p* < 0.006; *r* = 0.33) and tumor growth index (*p* < 0.002; *r* = 0.38), and CD68 expression also correlated with tumor size (*p* < 0.0001; *r* = 0.46) and tumor growth index (*p* < 0.0001, *r* = 0.48).

**Fig. 3:**
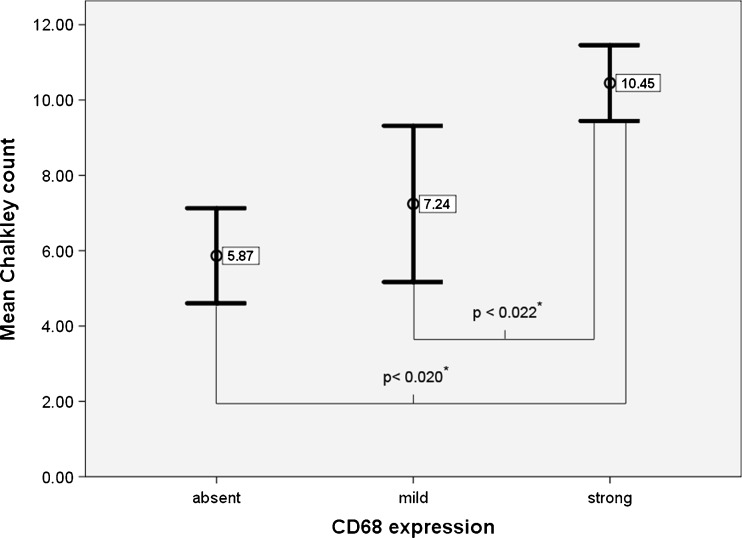
Relation between CD68 expression and microvessel density. The mean Chalkley count is significantly higher in tumors with strong CD68 expression. *Asterisk* denotes statistical differences calculated with the Scheffe test

The Chalkley count demonstrated a positive correlation with tumor size (*p* < 0.001; *r* = 0.40) and tumor growth index (*p* < 0,002; *r* = 0.37). Furthermore, the Chalkley count showed a significant correlation with the number of hemosiderin-positive cells (*p* < 0.003; *r* = 0.36), CD45-positive cells (*p* < 0.021 *r* = 0.28), and CD68-positive cells (*p* < 0.0001; *r* = 0.47). A one-way ANOVA and a Scheffe test confirmed the positive relation between the number of CD68-positive cells and microvessel density. Tumors with a high number of CD68-positive cells displayed a significantly higher microvascular density than tumors with low or no CD68-positive cells (Fig. 3).

In 61 patients, the tumors were morphologically classified based on their MRI appearance. Twenty-four tumors were classified as homogeneous, 8 as inhomogeneous, and 29 as cystic. Cystic and inhomogeneous tumors were significantly larger than homogeneous tumors (Table 3). Cystic and inhomogeneous tumors also displayed a significantly higher number of hemosiderin-positive cells than homogeneous tumors (Table 3).

**Table 3:** MRI appearance compared by hemosiderin deposition and size

MRI	Hemosiderin deposition^a^						Size^b^	
	Absent	Mild	Strong	*χ* ^2^	*df*	*p* value	Mean ± SD	*p* value
Homogeneous	15	7	2	8.67	2	<0.013	13.46 ± 8.44	<0.0001
Inhomogeneous/cystic	10	15	12				30.46 ± 8.43	

*MRI* magnetic resonance imaging

^a^Chi-square test

^b^Independent *t* test

No statistically significant correlations or differences were observed when patient age or duration of symptoms was taken into account.

## Discussion

To gain more insight into the mechanisms responsible for volume increase of vestibular schwannomas, possible correlations between histopathological markers and radiological and clinical characteristics of vestibular schwannomas were investigated. For studying the growth rate of a tumor, serial radiological observation is the preferred method. As most patients in this study were operated on shortly after diagnosis, in the majority of cases, only one preoperative MRI scan was obtained, which excluded this approach. As a surrogate, we used the growth index, which is only a rough estimate of the rate of tumor volume increase but allowed us to include a larger number of patients in the study.

To evaluate the role of proliferative activity in the volume increase of vestibular schwannomas, the cell cycle markers Ki-67 and histone H3 were used. Ki-67 as a parameter of growth of vestibular schwannomas has been studied by Niemczyk et al. [2], who compared clinically stable vestibular schwannomas with clinically growing cases. They found a significant difference in Ki-67 labeling index between the two groups. The mean labeling index in the growing tumors was 3.17 % compared to 1.11 % in the stable tumors. Overall, the Ki-67 index ranged from 0.22 to 5 %, with an average of 1.86 %. Gomez et al. [13] also investigated cell proliferation in vestibular schwannomas, but did not find a significant correlation between tumor growth and Ki-67 labeling index, which ranged from 0.2 to 2.2 %. We conclude that the labeling index of Ki-67 in our study (ranging from 0.1 to 1.8 %, with a mean of 0.6 %) is comparable with earlier published data.

Histone H3 as a proliferation marker has not been studied in vestibular schwannomas before. We did not find a correlation between the histone H3 labeling index and the tumor growth index. Taken together, our data and those from the literature indicate that cell proliferation is not a decisive factor in the expansion of vestibular schwannomas.

Degenerative changes such as cysts may contribute to tumor volume increase. Reports on the incidence of cyst formation in vestibular schwannomas vary between 5.7 and 48 %, with more recent studies indicating incidences of approximately 10 % [14–17]. Cystic tumors can display a relatively rapid increase in volume and generally become larger than noncystic tumors [18]. This also applies to our case series, the cystic and inhomogeneous tumors being significantly larger than the homogeneous tumors. The mechanisms responsible for cyst formation in vestibular schwannomas remain unclear. Gomez et al. [13] demonstrated a significant correlation between hemosiderin deposition, tumor size, and tumor heterogeneity and suggested that hemosiderin resorption might induce cyst formation. The same suggestion is made by Park et al. [19]. Our results are in line with these studies. We found significantly more hemosiderin deposition in cystic and inhomogeneous tumors compared to homogeneous tumors. The significant correlations between hemosiderin deposition and tumor size and tumor growth index also support this notion.

These results may offer a clue in the search for markers of tumor volume increase. Both cysts and iron deposition are radiologically demonstrable and might, therefore, constitute clinically applicable markers. A radiological study on pituitary macroadenomas by Stadlbauer et al. [20] described a significant difference in spectral signals measured in hemorrhagic versus nonhemorrhagic tumors. The authors suggested that the paramagnetic effect of hemosiderin deposition in hemorrhagic tumors on the MR images might provide an explanation. Using this approach to study iron deposition and cyst formation in vestibular schwannomas might establish a causal relationship between intratumoral hemorrhage and cyst formation.

Although vestibular schwannomas are relatively slow-growing neoplasms, they depend for further growth on a functional vascular system, as any other tumor [21]. The positive correlation between microvessel density and tumor size and tumor growth index we found is consistent with other published data [22, 23]. These findings suggest that angiogenesis may be important for the volume increase of vestibular schwannomas. Determining the degree of vascularization of vestibular schwannomas might also be possible using MRI diffusion or perfusion techniques [24–26]. Further research into this hypothesis could lead to another clinically applicable marker of tumor volume increase. These results also suggest that antiangiogenesis therapy might contribute to controlling tumor growth.

The degree of inflammation measured by the expression of CD68 and CD45 showed a positive significant correlation with tumor size and tumor growth index. Similar correlations have not been reported in the literature yet. In a study performed by Brieger el al. [27] on angiogenic growth factors in vestibular schwannomas, tumor-infiltrating CD68-positive lymphocytes were not detected. Labit et al. [28] only found a correlation between the number of CD45-positive cells and the duration of symptoms. The mechanism responsible for or the significance of an inflammatory reaction in vestibular schwannomas has not been elucidated as yet. Studies on breast cancer have addressed the role of the inflammatory microenvironment in tumor progression [29–35]. It has been proposed that inflammation which triggers angiogenesis might contribute to tumor progression. Macrophage activity is a major determinant of the intratumoral inflammatory milieu but other components of the inflammatory infiltrate also seem to modulate tumor behavior [36]. The significant associations between the degree of inflammation (i.e., CD45 and CD68) and tumor size and tumor growth index and, in addition, the significant increase of microvessel density in tumors with a higher number of CD68-positive cells (Fig. 3) suggest that similar processes take place in vestibular schwannomas. Several approaches targeting macrophage activity are currently under investigation [37, 38], making additional research of the inflammatory process in vestibular schwannomas even more interesting. The first step would be further typing of inflammatory cells present in vestibular schwannomas. Furthermore, their activation state and relationship with angiogenic growth factors should be examined.

The results of this study indicate that tumor volume increase of vestibular schwannomas is not based on cell proliferation alone. Contributing factors are intratumoral hemorrhage, vascularization, and degree of inflammation.

## Electronic supplementary material

Below is the link to the electronic supplementary material.

Supplementary Material Fig. 1 T1 weighted gadolinium enhanced MRI image of a cystic vestibular schwannoma (JPEG 177 kb)

High Resolution image (TIFF 3173 kb)

Supplementary Material Fig. 2 T1 weighted gadolinium enhanced MRI image of a homogeneous vestibular schwannoma (JPEG 176 kb)

High Resolution image (TIFF 3172 kb)

Supplementary Material Fig. 3 T1 weighted gadolinium enhanced MRI image of an inhomogeneous vestibular schwannoma (JPEG 142 kb)

High Resolution image (TIFF 3173 kb)

Supplementary Material Fig. 4 Scattergrams of correlations between maximum tumor diameter and **A** CD45 expression, **B** CD68 expression, **C** hemosiderin deposition and **D** microvessel density. *p < 0.05, **p < 0.01 (JPEG 98 kb)

High Resolution image (TIFF 1356 kb)

Supplementary Material Fig. 5 Scattergrams of correlations between tumor growth index and **A** CD45 expression, **B** CD68 expression, **C** hemosiderin deposition and **D** microvessel density. *p < 0.05, **p < 0.01 (JPEG 100 kb)

High Resolution image (TIFF 1335 kb)

Supplementary Material Fig. 6 Scattergrams of correlations between microvessel density and **A** CD45 expression, **B** CD68 expression and **C** hemosiderin deposition. *p < 0.05, **p < 0.01 (JPEG 76 kb)

High Resolution image (TIFF 1287 kb)

ESM 1 (DOC 94 kb)

## References

[1] Szeremeta W, Monsell EM, Rock JP, Caccamo DV (1995). Proliferation indices of vestibular schwannomas by Ki-67 and proliferating cell nuclear antigen. Am J Otol.

[2] Niemczyk K, Vaneecloo FM, Lecomte MH, Lejeune JP, Lemaitre L, Skarzynski H (2000). Correlation between Ki-67 index and some clinical aspects of acoustic neuromas (vestibular schwannomas). Otolaryngol Head Neck Surg.

[3] Davidson EJ, Morris LS, Scott IS, Rushbrook SM, Bird K, Laskey RA (2003). Minichromosome maintenance (Mcm) proteins, cyclin B1 and D1, phosphohistone H3 and in situ DNA replication for functional analysis of vulval intraepithelial neoplasia. Br J Cancer.

[4] Brenner RM, Slayden OD, Rodgers WH, Critchley HO, Carroll R, Nie XJ (2003). Immunocytochemical assessment of mitotic activity with an antibody to phosphorylated histone H3 in the macaque and human endometrium. Hum Reprod.

[5] Takahashi H, Murai Y, Tsuneyama K, Nomoto K, Okada E, Fujita H (2006). Overexpression of phosphorylated histone H3 is an indicator of poor prognosis in gastric adenocarcinoma patients. Appl Immunohistochem Mol Morphol.

[6] (1995) Committee on Hearing and Equilibrium guidelines for the evaluation of hearing preservation in acoustic neuroma (vestibular schwannoma). American Academy of Otolaryngology-Head and Neck Surgery Foundation, INC. Otolaryngol Head Neck Surg 113(3):179–180. 10.1016/S0194-5998(95)70101-X 7675475

[7] Bovee JV, van den Broek LJ, de Boer WI, Hogendoorn PC (1998). Expression of growth factors and their receptors in adamantinoma of long bones and the implication for its histogenesis. J Pathol.

[8] Vermeulen PB, Gasparini G, Fox SB, Colpaert C, Marson LP, Gion M (2002). Second international consensus on the methodology and criteria of evaluation of angiogenesis quantification in solid human tumours. Eur J Cancer.

[9] Fox SB, Leek RD, Weekes MP, Whitehouse RM, Gatter KC, Harris AL (1995). Quantitation and prognostic value of breast cancer angiogenesis: comparison of microvessel density, Chalkley count, and computer image analysis. J Pathol.

[10] de Andrea CE, Wiweger MI, Bovee JV, Romeo S, Hogendoorn PC (2011). Peripheral chondrosarcoma progression is associated with increased type X collagen and vascularisation. Virchows Arch.

[11] Koutsimpelas D, Stripf T, Heinrich UR, Mann WJ, Brieger J (2007). Expression of vascular endothelial growth factor and basic fibroblast growth factor in sporadic vestibular schwannomas correlates to growth characteristics. Otol Neurotol.

[12] Bedavanija A, Brieger J, Lehr HA, Maurer J, Mann WJ (2003). Association of proliferative activity and size in acoustic neuroma: implications for timing of surgery. J Neurosurg.

[13] Gomez-Brouchet A, Delisle MB, Cognard C, Bonafe A, Charlet JP, Deguine O (2001). Vestibular schwannomas: correlations between magnetic resonance imaging and histopathologic appearance. Otol Neurotol.

[14] Jeng CM, Huang JS, Lee WY, Wang YC, Kung CH, Lau MK (1995). Magnetic resonance imaging of acoustic schwannomas. J Formos Med Assoc.

[15] Fundova P, Charabi S, Tos M, Thomsen J (2000). Cystic vestibular schwannoma: surgical outcome. J Laryngol Otol.

[16] Jones SE, Baguley DM, Moffat DA (2007). Are facial nerve outcomes worse following surgery for cystic vestibular schwannoma?. Skull Base.

[17] Sinha S, Sharma BS (2008). Cystic acoustic neuromas: surgical outcome in a series of 58 patients. J Clin Neurosci.

[18] Charabi S, Mantoni M, Tos M, Thomsen J (1994). Cystic vestibular schwannomas: neuroimaging and growth rate. J Laryngol Otol.

[19] Park CK, Kim DC, Park SH, Kim JE, Paek SH, Kim DG (2006). Microhemorrhage, a possible mechanism for cyst formation in vestibular schwannomas. J Neurosurg.

[20] Stadlbauer A, Buchfelder M, Nimsky C, Saeger W, Salomonowitz E, Pinker K (2008). Proton magnetic resonance spectroscopy in pituitary macroadenomas: preliminary results. J Neurosurg.

[21] Folkman J (1971). Tumor angiogenesis: therapeutic implications. N Engl J Med.

[22] Charabi S, Simonsen K, Charabi B, Jacobsen GK, Moos T, Rygaard J (1996). Nerve growth factor receptor expression in heterotransplanted vestibular schwannoma in athymic nude mice. Acta Otolaryngol.

[23] Charabi S (1997). Acoustic neuroma/vestibular schwannoma in vivo and in vitro growth models. A clinical and experimental study. Acta Otolaryngol Suppl.

[24] van Rijswijk CS, Kunz P, Hogendoorn PC, Taminiau AH, Doornbos J, Bloem JL (2002). Diffusion-weighted MRI in the characterization of soft-tissue tumors. J Magn Reson Imaging.

[25] Bonneville F, Savatovsky J, Chiras J (2007). Imaging of cerebellopontine angle lesions: an update. Part 2: intra-axial lesions, skull base lesions that may invade the CPA region, and non-enhancing extra-axial lesions. Eur Radiol.

[26] Pedrosa I, Alsop DC, Rofsky NM (2009). Magnetic resonance imaging as a biomarker in renal cell carcinoma. Cancer.

[27] Brieger J, Bedavanija A, Lehr HA, Maurer J, Mann WJ (2003). Expression of angiogenic growth factors in acoustic neurinoma. Acta Otolaryngol.

[28] Labit-Bouvier C, Crebassa B, Bouvier C, Andrac-Meyer L, Magnan J, Charpin C (2000). Clinicopathologic growth factors in vestibular schwannomas: a morphological and immunohistochemical study of 69 tumours. Acta Otolaryngol.

[29] Buddingh EP, Kuijjer ML, Duim RA, Burger H, Agelopoulos K, Myklebost O (2011). Tumor-infiltrating macrophages are associated with metastasis suppression in high-grade osteosarcoma: a rationale for treatment with macrophage activating agents. Clin Cancer Res.

[30] Colotta F, Allavena P, Sica A, Garlanda C, Mantovani A (2009). Cancer-related inflammation, the seventh hallmark of cancer: links to genetic instability. Carcinogenesis.

[31] Hsu HP, Shan YS, Lai MD, Lin PW (2010). Osteopontin-positive infiltrating tumor-associated macrophages in bulky ampullary cancer predict survival. Cancer Biol Ther.

[32] Lin EY, Nguyen AV, Russell RG, Pollard JW (2001). Colony-stimulating factor 1 promotes progression of mammary tumors to malignancy. J Exp Med.

[33] Mantovani A, Romero P, Palucka AK, Marincola FM (2008). Tumour immunity: effector response to tumour and role of the microenvironment. Lancet.

[34] Ojalvo LS, Whittaker CA, Condeelis JS, Pollard JW (2010). Gene expression analysis of macrophages that facilitate tumor invasion supports a role for Wnt-signaling in mediating their activity in primary mammary tumors. J Immunol.

[35] Qian B, Deng Y, Im JH, Muschel RJ, Zou Y, Li J (2009). A distinct macrophage population mediates metastatic breast cancer cell extravasation, establishment and growth. PLoS One.

[36] Solinas G, Germano G, Mantovani A, Allavena P (2009). Tumor-associated macrophages (TAM) as major players of the cancer-related inflammation. J Leukoc Biol.

[37] Allavena P, Signorelli M, Chieppa M, Erba E, Bianchi G, Marchesi F (2005). Anti-inflammatory properties of the novel antitumor agent yondelis (trabectedin): inhibition of macrophage differentiation and cytokine production. Cancer Res.

[38] Mukhtar RA, Nseyo O, Campbell MJ, Esserman LJ (2011). Tumor-associated macrophages in breast cancer as potential biomarkers for new treatments and diagnostics. Expert Rev Mol Diagn.

