# Supplementary figures and images for: Intratumoral hemorrhage, vessel density, and the inflammatory reaction contribute to volume increase of sporadic vestibular schwannomas
Source: Virchows Arch. 2012 May 4;460(6):629–36. doi: 10.1007/s00428-012-1236-9 (PMC3371334; doi:10.1007/s00428-012-1236-9)

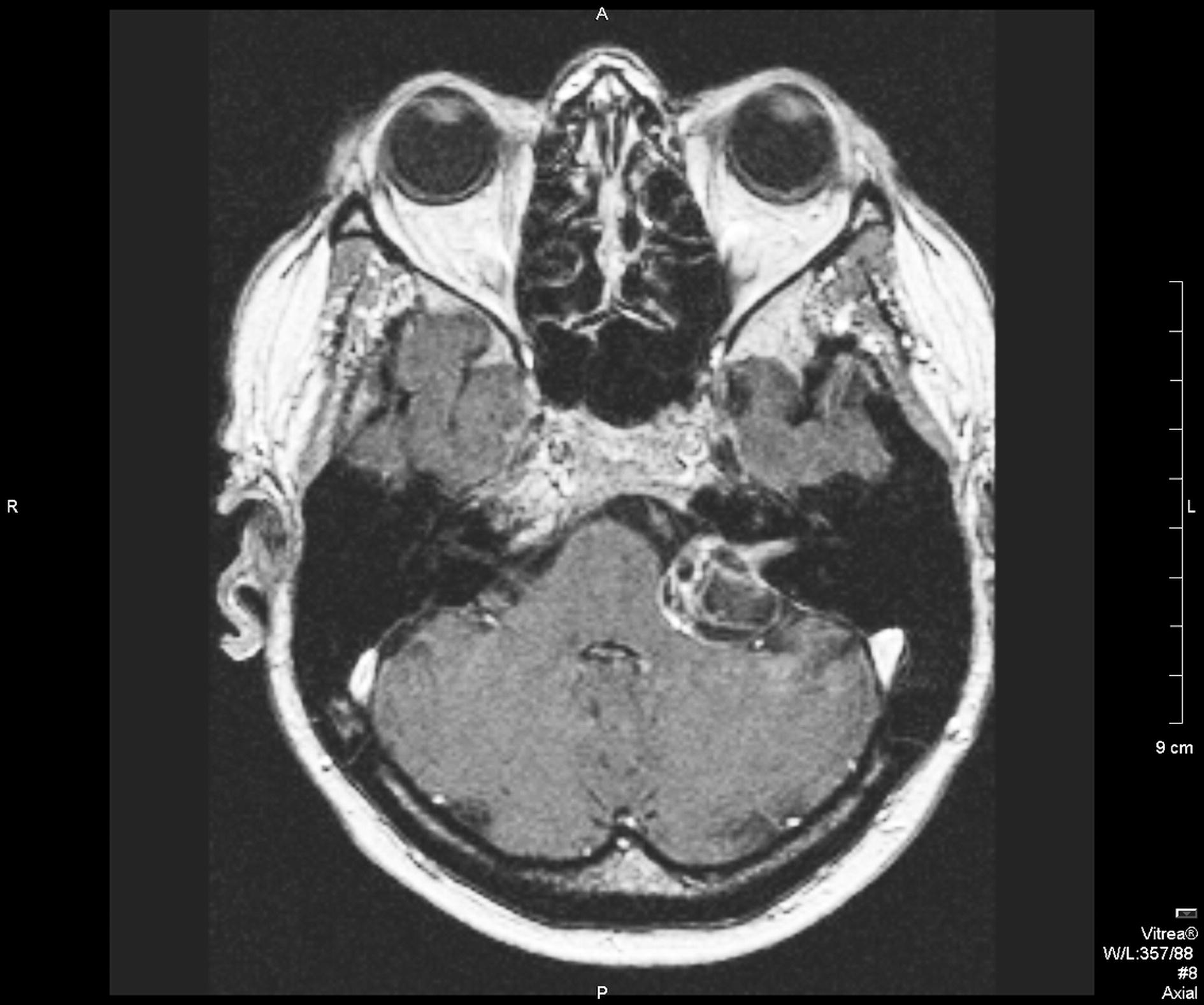

Supplement: Supplementary file 1 — T1 weighted gadolinium enhanced MRI image of a cystic vestibular schwannoma (JPEG 177 kb) [file 428_2012_1236_Fig4_ESM.jpg]

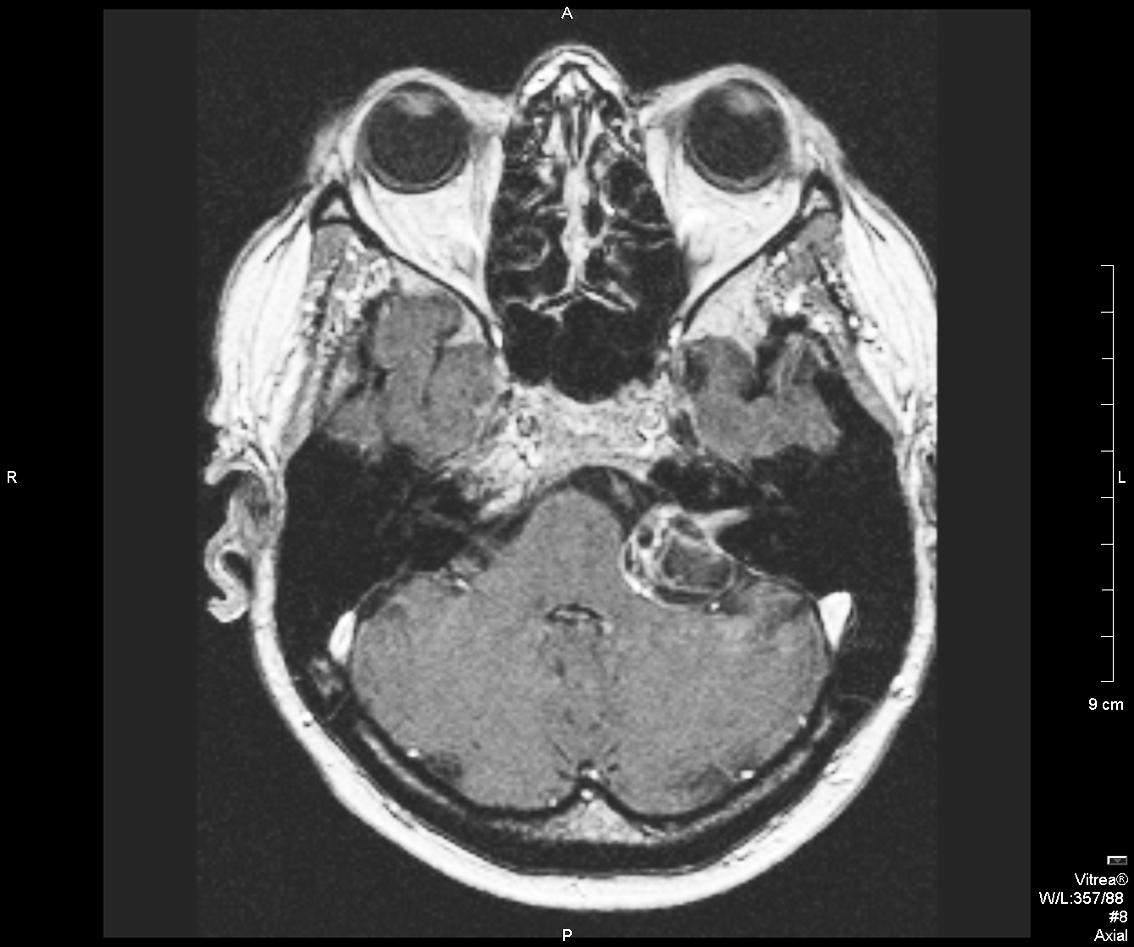

Supplement: Supplementary file 2 — High Resolution image (TIFF 3173 kb) [file 428_2012_1236_MOESM1_ESM.tif]

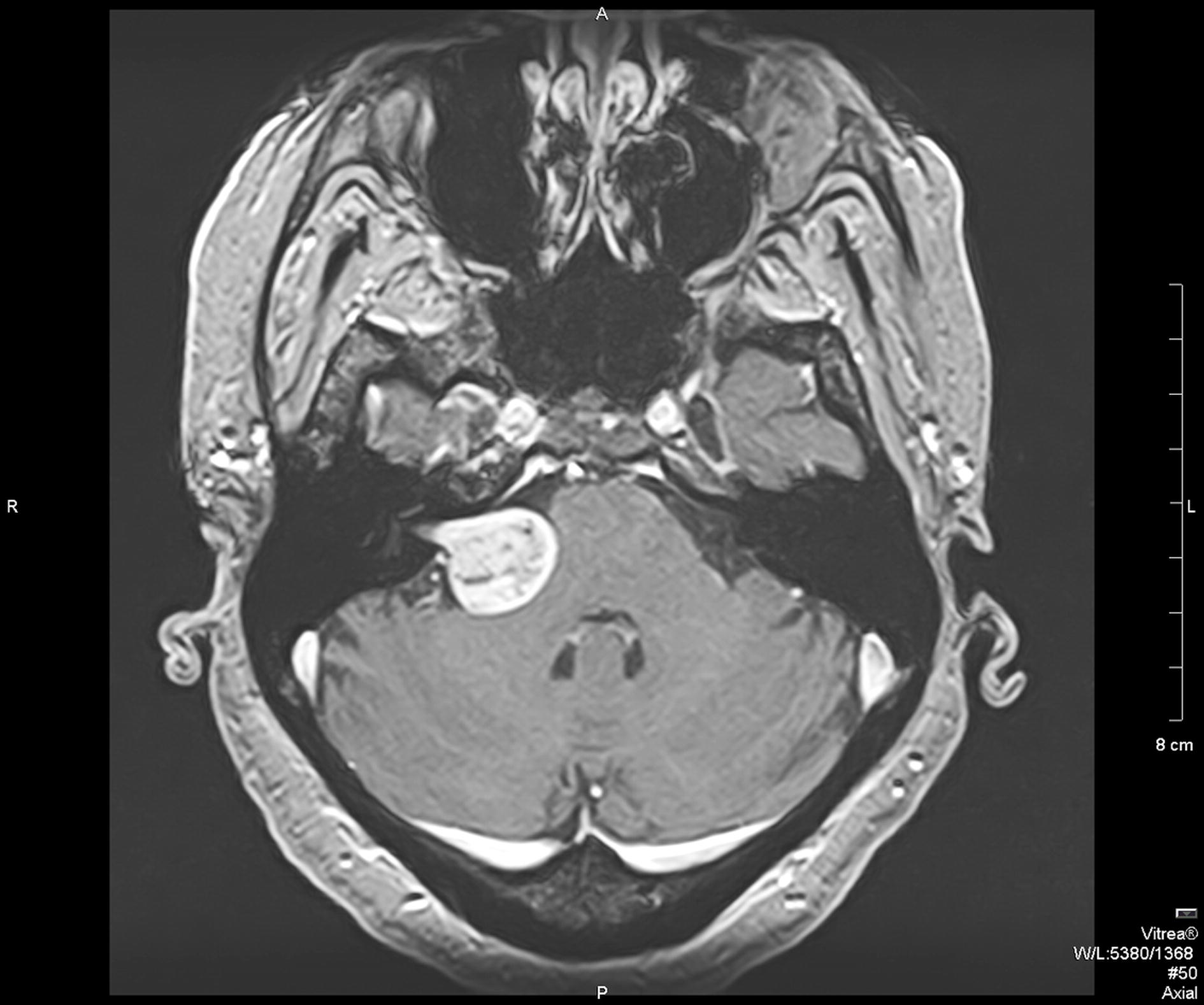

Supplement: Supplementary file 3 — T1 weighted gadolinium enhanced MRI image of a homogeneous vestibular schwannoma (JPEG 176 kb) [file 428_2012_1236_Fig5_ESM.jpg]

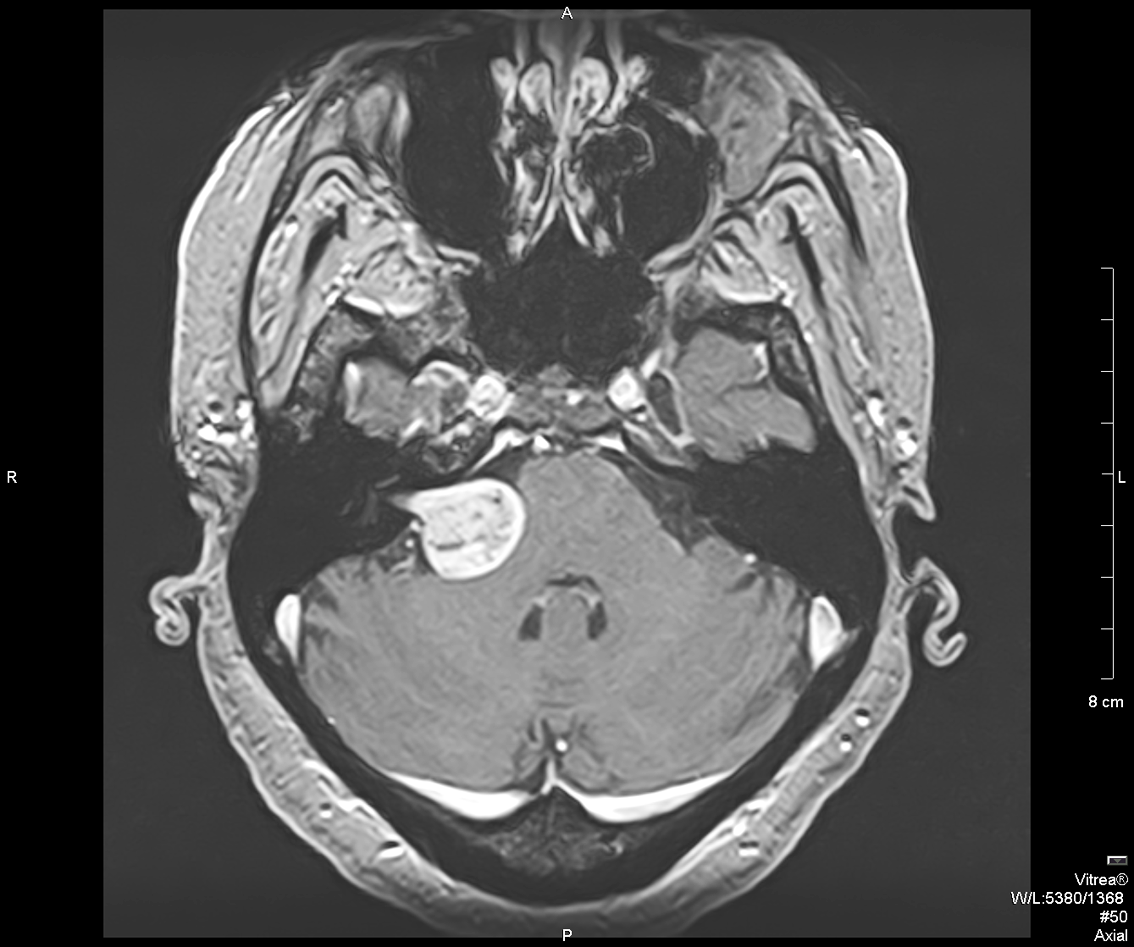

Supplement: Supplementary file 4 — High Resolution image (TIFF 3172 kb) [file 428_2012_1236_MOESM2_ESM.tif]

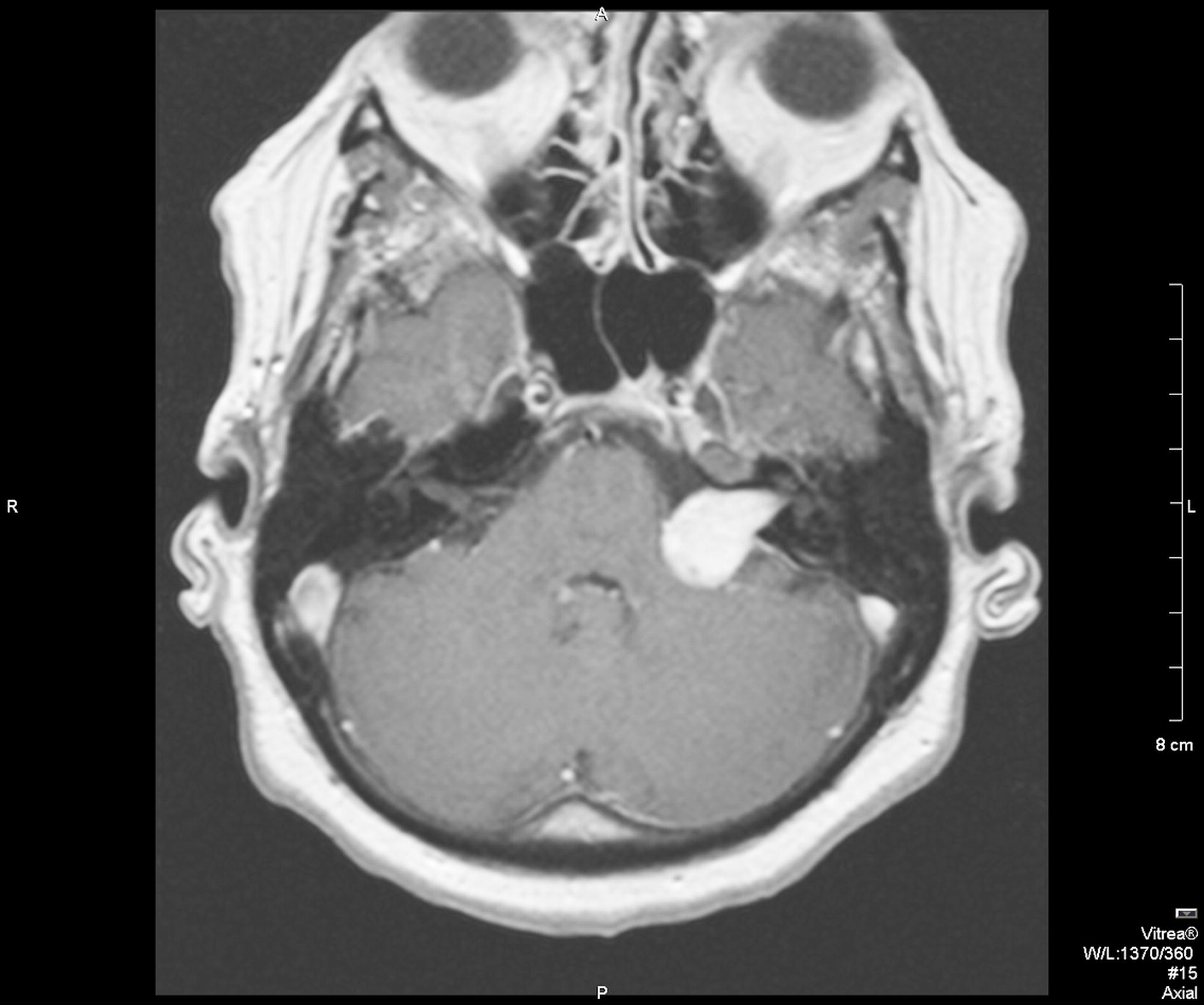

Supplement: Supplementary file 5 — T1 weighted gadolinium enhanced MRI image of an inhomogeneous vestibular schwannoma (JPEG 142 kb) [file 428_2012_1236_Fig6_ESM.jpg]

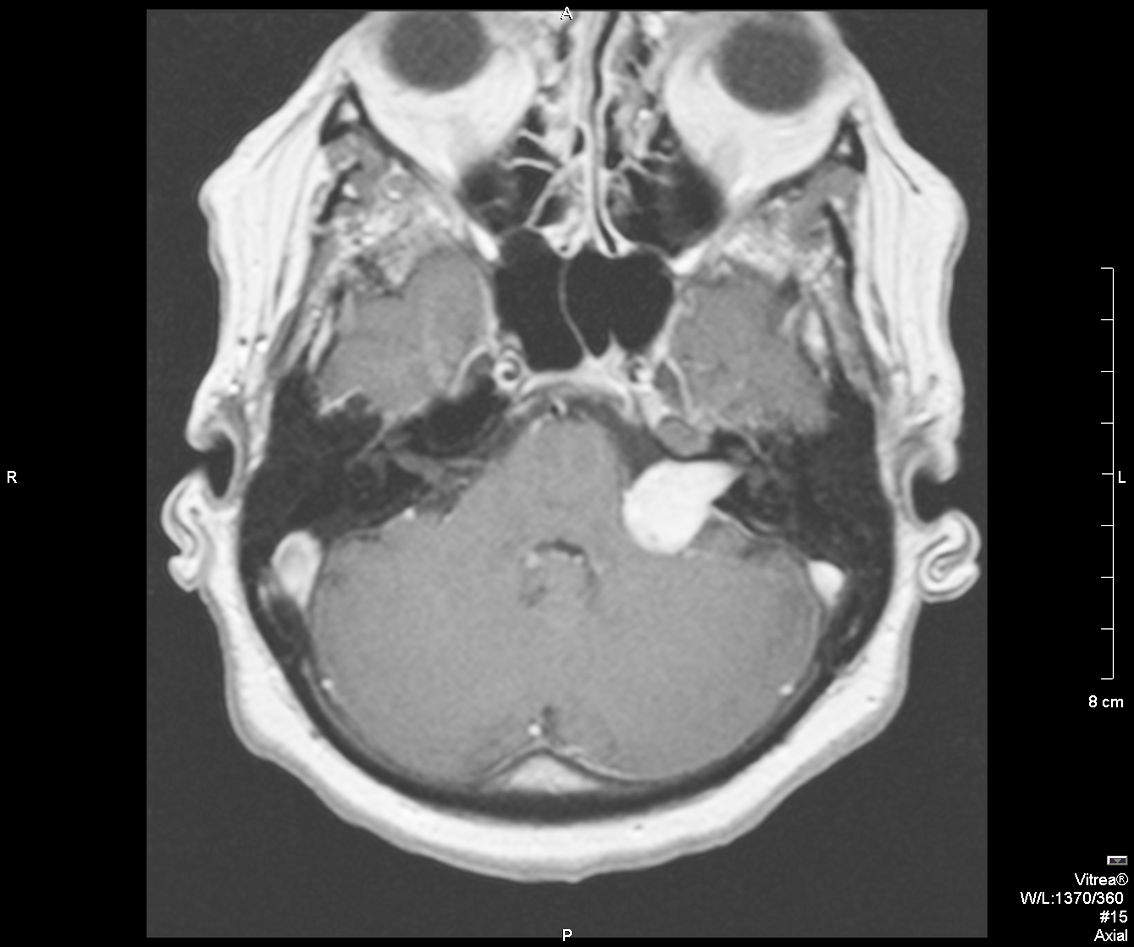

Supplement: Supplementary file 6 — High Resolution image (TIFF 3173 kb) [file 428_2012_1236_MOESM3_ESM.tif]

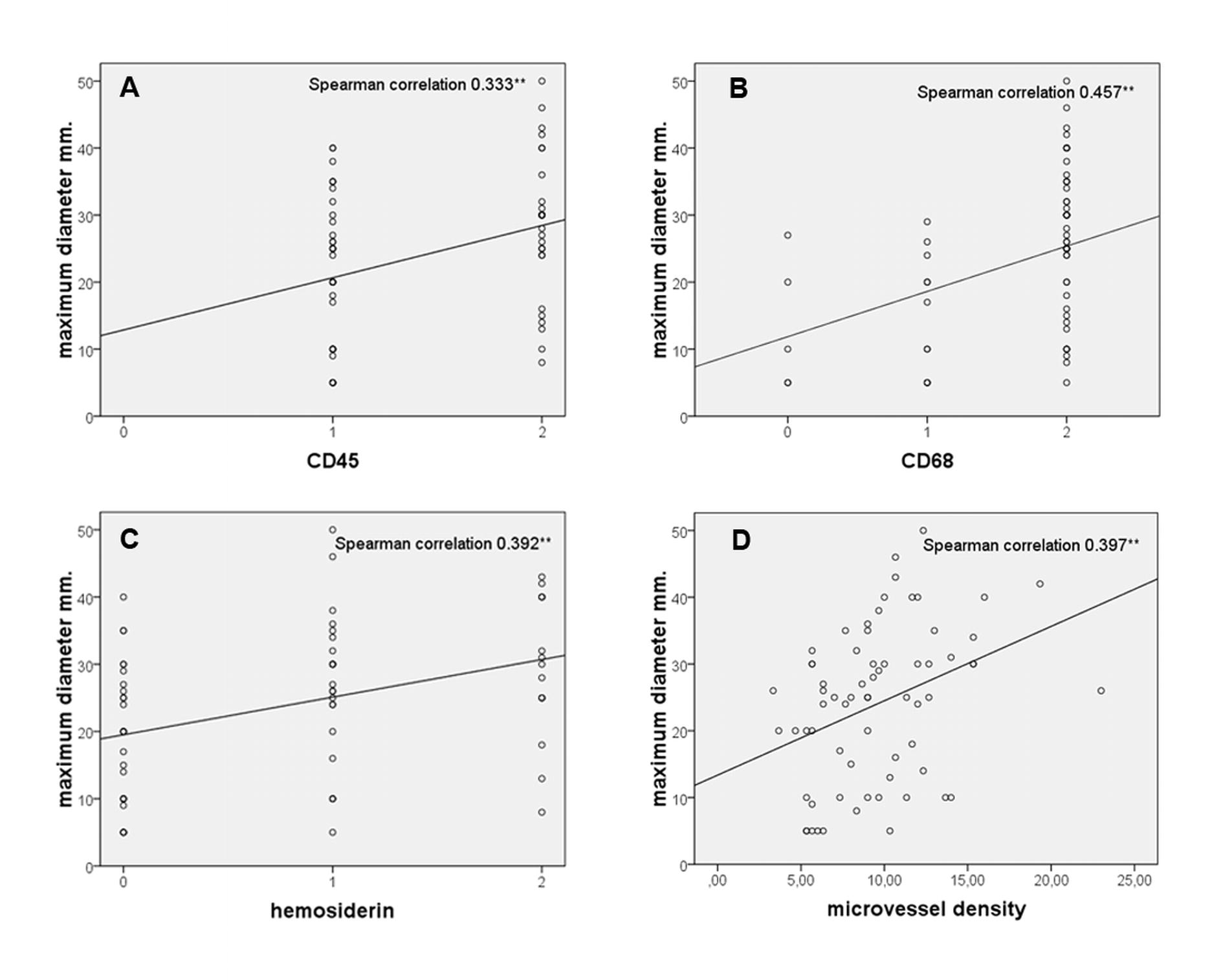

Supplement: Supplementary file 7 — Scattergrams of correlations between maximum tumor diameter and A CD45 expression, B CD68 expression, C hemosiderin deposition and D microvessel density. *p < 0.05, **p < 0.01 (JPEG 98 kb) [file 428_2012_1236_Fig7_ESM.jpg]

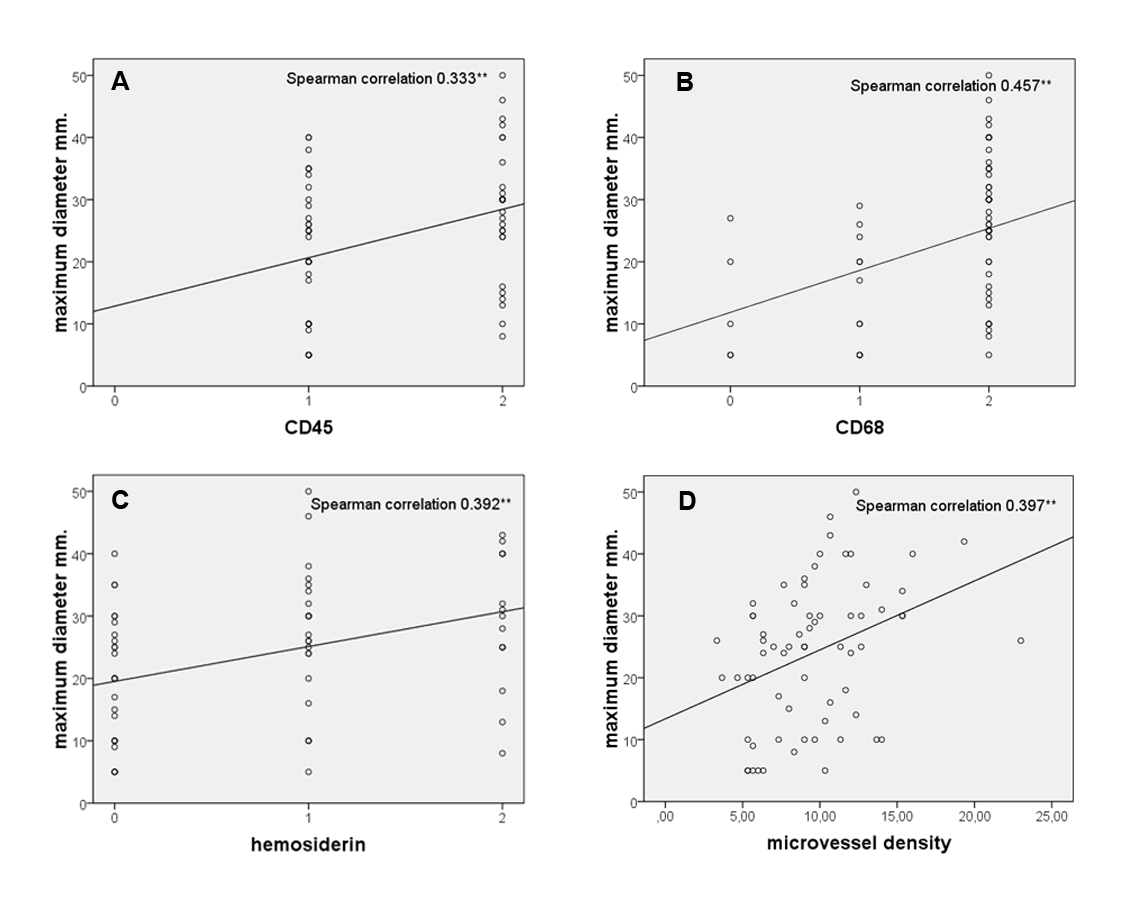

Supplement: Supplementary file 8 — High Resolution image (TIFF 1356 kb) [file 428_2012_1236_MOESM4_ESM.tif]

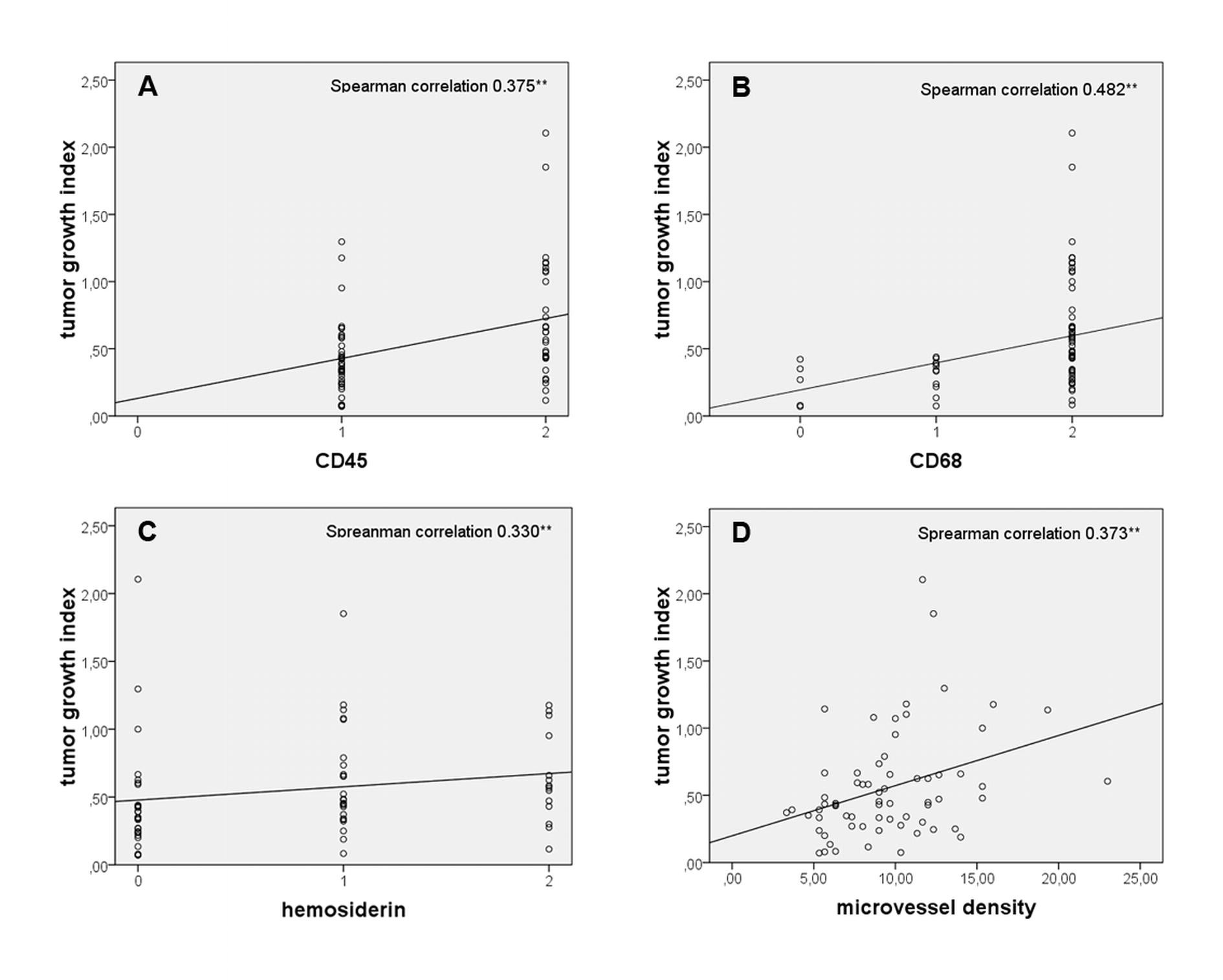

Supplement: Supplementary file 9 — Scattergrams of correlations between tumor growth index and A CD45 expression, B CD68 expression, C hemosiderin deposition and D microvessel density. *p < 0.05, **p < 0.01 (JPEG 100 kb) [file 428_2012_1236_Fig8_ESM.jpg]

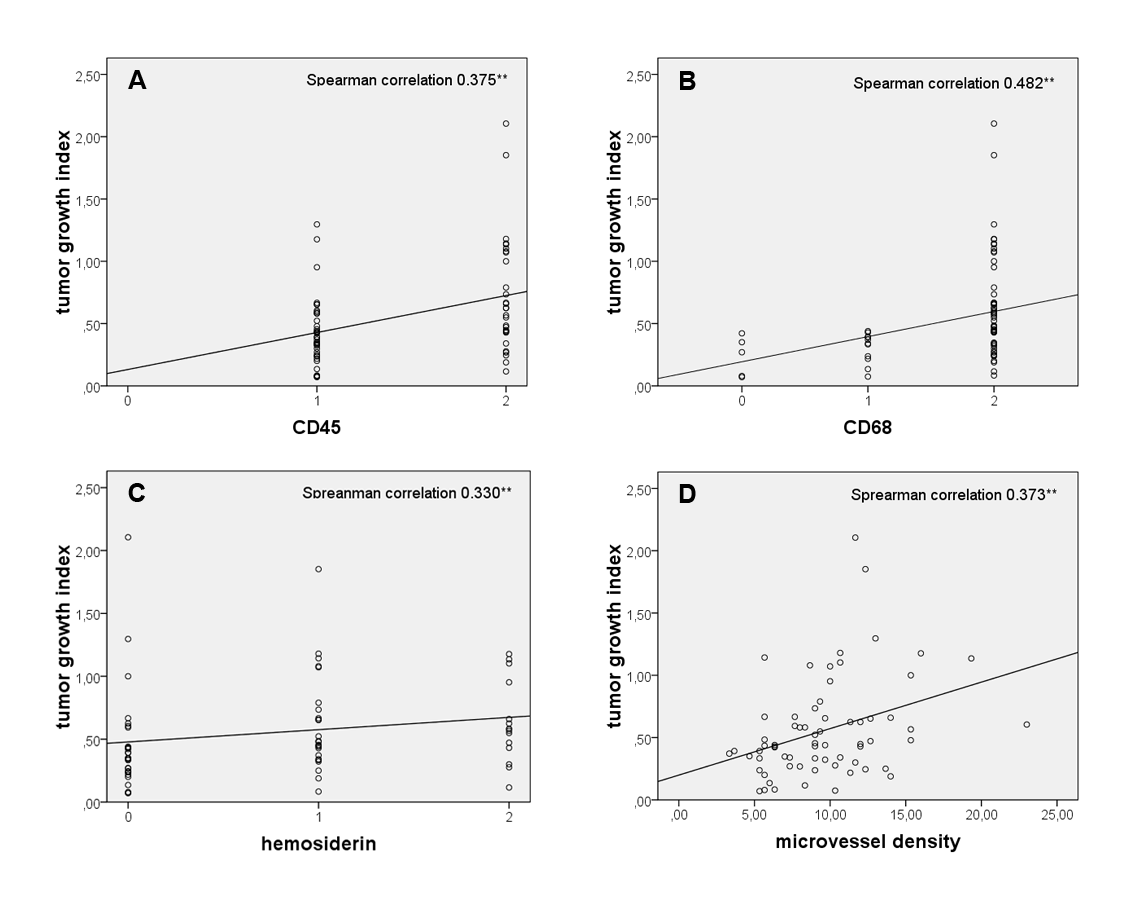

Supplement: Supplementary file 10 — High Resolution image (TIFF 1335 kb) [file 428_2012_1236_MOESM5_ESM.tif]

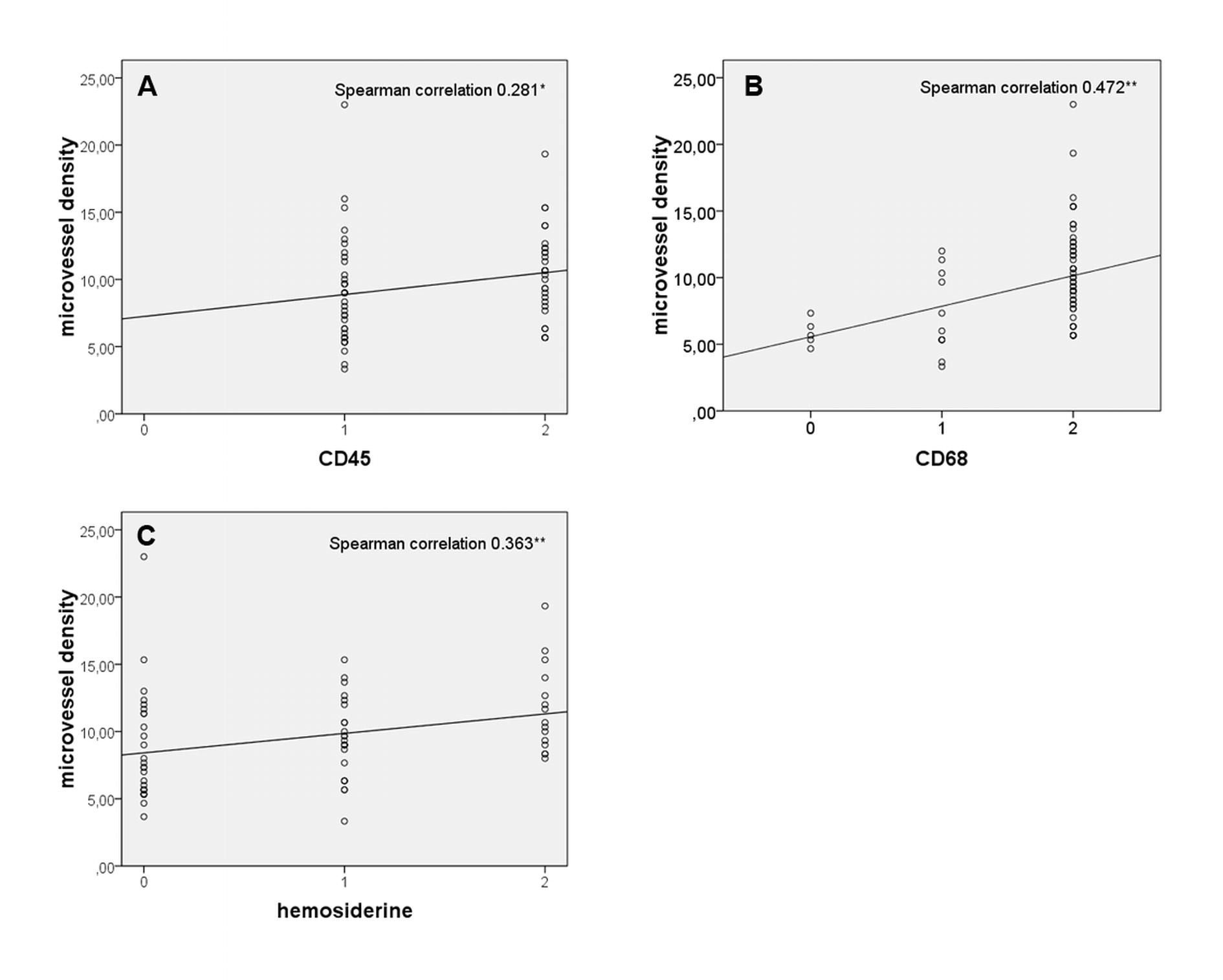

Supplement: Supplementary file 11 — Scattergrams of correlations between microvessel density and A CD45 expression, B CD68 expression and C hemosiderin deposition. *p < 0.05, **p < 0.01 (JPEG 76 kb) [file 428_2012_1236_Fig9_ESM.jpg]

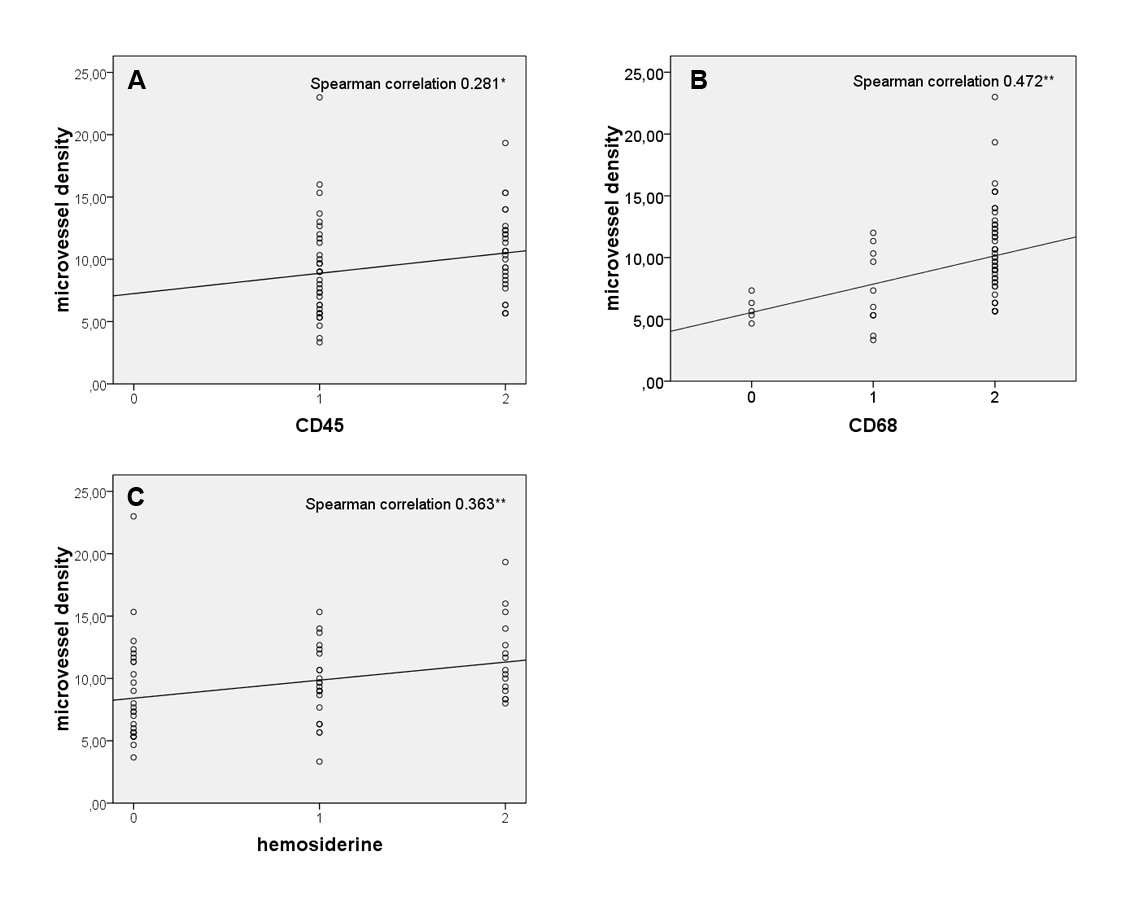

Supplement: Supplementary file 12 — High Resolution image (TIFF 1287 kb) [file 428_2012_1236_MOESM6_ESM.tif]
