# Supplementary material for: Intratumoral hemorrhage, vessel density, and the inflammatory reaction contribute to volume increase of sporadic vestibular schwannomas
Source: Virchows Arch. 2012 May 4;460(6):629–36. doi: 10.1007/s00428-012-1236-9 (PMC3371334; doi:10.1007/s00428-012-1236-9)
Supplement: Supplementary file 13 — (DOC 94 kb) [file 428_2012_1236_MOESM7_ESM.doc]

| **Pat.** | **sex** | **age** | **MRI appearance** | **Size** | **Tumor growth index** |
| --- | --- | --- | --- | --- | --- |
| 1 | f | 59 | homogeneous | 35 | 0,59 |
| 2 | f | 37 | cystic | 10 | 0,27 |
| 3 | f | 46 | inhomogeneous | 30 | 0,65 |
| 4 | f | 71 | homogeneous | 5 | 0,07 |
| 5 | m | 34 | cystic | 40 | 1,18 |
| 6 | f | 15 | homogeneous | 5 | 0,33 |
| 7 | m | 56 | homogeneous | 24 | 0,43 |
| 8 | f | 63 | homogeneous | 5 | 0,08 |
| 9 | f | 51 | cystic | 28 | 0,55 |
| 10 | m | 60 | cystic | 20 | 0,33 |
| 11 | f | 28 | inhomogeneous | 32 | 1,14 |
| 12 | m | 51 | cystic | 20 | 0,39 |
| 13 | m | 42 | inhomogeneous | 40 | 0,95 |
| 14 | m | 31 | homogeneous | 10 | 0,32 |
| 15 | m | 59 | cystic | 26 | 0,44 |
| 16 | m | 53 | cystic | 30 | 0,57 |
| 17 | f | 55 | x | 25 | 0,45 |
| 18 | f | 57 | homogeneous | 20 | 0,35 |
| 19 | m | 42 | homogeneous | 10 | 0,24 |
| 20 | f | 69 | homogeneous | 8 | 0,12 |
| 21 | f | 45 | homogeneous | 9 | 0,20 |
| 22 | f | 64 | inhomogeneous | 40 | 0,63 |
| 23 | m | 40 | inhomogeneous | 25 | 0,63 |
| 24 | f | 62 | cystic | 30 | 0,48 |
| 25 | f | 58 | cystic | 38 | 0,66 |
| 26 | m | 69 | cystic | 30 | 0,43 |
| 27 | m | 57 | homogeneous | 14 | 0,25 |
| 28 | m | 30 | homogeneous | 20 | 0,67 |
| 29 | m | 58 | cystic | 25 | 0,43 |
| 30 | m | 37 | cystic | 42 | 1,14 |
| 31 | m | 72 | cystic | 25 | 0,35 |
| 32 | f | 47 | inhomogeneous | 31 | 0,66 |
| 33 | m | 37 | homogeneous | 5 | 0,14 |
| 34 | f | 71 | cystic | 34 | 0,48 |
| 35 | f | 38 | inhomogeneous | 30 | 0,79 |
| 36 | m | 47 | inhomogeneous | 13 | 0,28 |
| 37 | f | 66 | cystic | 29 | 0,44 |
| 38 | f | 60 | x | 18 | 0,30 |
| 39 | m | 43 | cystic | 25 | 0,58 |
| 40 | m | 42 | homogeneous | 10 | 0,24 |
| 41 | f | 56 | homogeneous | 15 | 0,27 |
| 42 | f | 53 | homogeneous | 25 | 0,47 |

| **Pat.** | **sex** | **age** | **MRI appearance** | **Size** | **Tumor growth index** |
| --- | --- | --- | --- | --- | --- |
| 43 | f | 64 | cystic | 27 | 0,42 |
| 44 | f | 67 | homogeneous | 5 | 0,07 |
| 45 | m | 55 | cystic | 32 | 0,58 |
| 46 | m | 36 | cystic | 24 | 0,67 |
| 47 | f | 56 | cystic | 24 | 0,43 |
| 48 | m | 46 | homogeneous | 10 | 0,22 |
| 49 | f | 70 | cystic | 26 | 0,37 |
| 50 | f | 67 | cystic | 30 | 0,45 |
| 51 | f | 50 | homogeneous | 17 | 0,34 |
| 52 | m | 67 | cystic | 35 | 0,52 |
| 53 | f | 43 | cystic | 26 | 0,60 |
| 54 | f | 60 | homogeneous | 5 | 0,08 |
| 55 | m | 27 | cystic | 50 | 1,85 |
| 56 | f | 47 | homogeneous | 16 | 0,34 |
| 57 | f | 27 | cystic | 35 | 1,30 |
| 58 | f | 25 | x | 27 | 1,08 |
| 59 | f | 39 | cystic | 43 | 1,10 |
| 60 | f | 19 | x | 40 | 2,11 |
| 61 | m | 49 | cystic | 36 | 0,73 |
| 62 | f | 51 | x | 20 | 0,39 |
| 63 | f | 40 | homogeneous | 10 | 0,25 |
| 64 | f | 30 | x | 30 | 1,00 |
| 65 | f | 53 | homogeneous | 10 | 0,19 |
| 66 | f | 39 | cystic | 46 | 1,18 |
| 67 | f | 28 | homogeneous | 30 | 1,07 |
